# Coconut (*Cocos nucifera*) tree disease dataset: A dataset for disease detection and classification for machine learning applications

**DOI:** 10.1016/j.dib.2023.109690

**Published:** 2023-10-15

**Authors:** Sandip Thite, Yogesh Suryawanshi, Kailas Patil, Prawit Chumchu

**Affiliations:** a Vishwakarma University, Pune, India; b Kasetsart University, Sriracha, Thailand

**Keywords:** Coconut tree diseases, Disease detection, Disease classification, Machine learning

## Abstract

The ``Coconut (*Cocos nucifera*) Tree Disease Dataset'' comprises 5,798 images across five disease categories: ``Bud Root Dropping,'' ``Bud Rot,'' ``Gray Leaf Spot,'' ``Leaf Rot,'' and ``Stem Bleeding.'' This dataset is intended for machine learning applications, facilitating disease detection and classification in coconut trees. The dataset's diversity and size make it suitable for training and evaluating disease detection models. The availability of this dataset will support advancements in plant pathology and aid in the sustainable management of coconut plantations. By providing a valuable resource for researchers, this dataset contributes to improved disease management and sustainable coconut plantation practices.

Specifications Table

**Table:** 

Subject	Applied Machine Learning, Agriculture
Specific subject area	Agronomy & Crop Science
Data format	Raw
Type of data	Images
Description of data collection	The data collection process for the "Coconut Tree Disease Dataset" was meticulously carried out in the Kendur region, located in Taluka- Shirur, Pune district, Maharashtra, India. To ensure the dataset's relevance and diversity, images were collected from various coconut plantations in the Kendur region, considering different growth stages, environmental conditions, and disease manifestations.
Data source location	**Kendur, Taluka- Shirur, Dist -Pune** Pin - 412403. Maharashtra, Country- India. Latitude- 18°47′06.4"N, Longitude- 74°01′19.5"E
Data accessibility	Repository name: Coconut Tree Disease Dataset Data identification number: 10.17632/gh56wbsnj5.1 Direct URL to data: https://data.mendeley.com/datasets/gh56wbsnj5/1

## 1 Value of the Data

• The “Coconut Tree Disease Dataset” is highly relevant to the agricultural and plant pathology research communities. It addresses an important real-world problem - coconut tree diseases - which can have severe implications for the coconut industry and food security.
• First Open-Access Dataset: This dataset is the first openly accessible collection of coconut tree diseases samples. It facilitates collaboration among researchers, accelerating advancements in disease detection, monitoring, and management in coconut cultivation.
• With 5,798 images representing five distinct disease categories, the dataset exhibits diversity and comprehensiveness. Such diversity is crucial for training and evaluating machine learning algorithms, ensuring their effectiveness in detecting and classifying different coconut tree diseases..
• Machine Learning Applications: The availability of this dataset opens avenues for researchers to develop and compare advanced machine learning models for coconut tree disease detection and classification. It encourages innovative approaches and fosters collaborations in the field of plant pathology.

## 2 Data Description

The image datasets play a crucial role in various fields, ranging from computer vision and machine learning to medical research and social sciences [[1], [2], [3], [4], [5]], [6],^17^]. The objective of the ``Coconut (*Cocos nucifera*) Tree Disease Dataset'' is to provide a high-quality and diverse collection of images for facilitating machine learning applications in automated disease detection and classification in coconut (*Cocos nucifera*) trees. The ``Coconut Tree Disease Dataset'' contains a collection of high-resolution images, each with dimensions of 768 pixels in width and 1024 pixels in height. The images have a resolution of 72 dots per inch (dpi), ensuring clarity and detail in the visual representation of the coconut tree diseases.

Each image in the dataset is associated with one of the five disease classes, providing a well-balanced representation of coconut tree diseases (Table 1). The study of ``Bud Root Dropping,'' ``Bud Rot,'' ``Gray Leaf Spot,'' ``Leaf Rot,'' and ``Stem Bleeding'' in coconut trees holds paramount importance due to their collective impact on the overall health and productivity of coconut plantations. Each of these diseases presents unique challenges and potential threats to the coconut industry. ``Bud Root Dropping'' affects early growth stages, potentially leading to stunted development and reduced yield. ``Bud Rot,'' a fungal disease, can swiftly devastate entire groves if not promptly identified and managed. ``Gray Leaf Spot'' poses a significant threat, as it spreads rapidly and can lead to widespread defoliation. ``Leaf Rot'' compromises the photosynthetic capacity of the tree, directly impacting its vitality. ``Stem Bleeding'' is a chronic condition, progressively weakening the tree's structural integrity. A comprehensive understanding of these diseases is essential for implementing targeted control measures, preventing widespread outbreaks, and ultimately ensuring the long-term viability of coconut plantations. The dataset we present here is a crucial resource for researchers and practitioners dedicated to combatting these specific diseases, enabling them to develop more effective and tailored solutions for disease management in coconut trees [18,19].

**Table 1:** Sample images of coconut disease dataset.

**Fig. 1:**
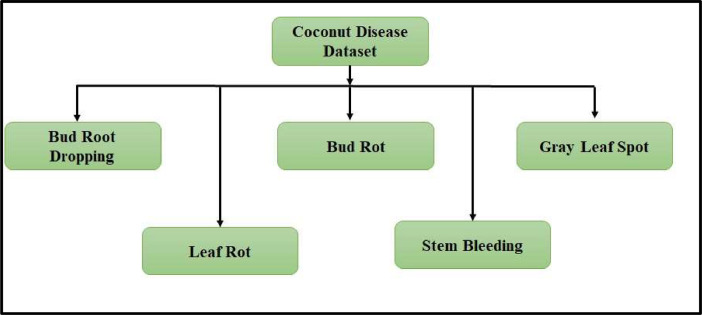
Directory structure of the coconut tree disease dataset.

The dataset was captured using the rear cameras of Samsung F23 5G Mobile, which boasts high-resolution imaging capabilities at Kendur, Maharashtra (18°47′06.4"N 74°01′19.5"E). The use of mobile cameras for data collection allows for flexibility and ease of capturing images directly in the coconut plantations, ensuring real-world representations of the diseases in their natural settings. The dataset's high-resolution and mobile camera origin contribute to the dataset's quality and authenticity, making it a valuable resource for researchers and practitioners interested in developing advanced disease detection and classification models. Each category is labelled and organized in separate folders, ensuring easy access and identification of specific disease samples. Fig. 1 shows directory structure of the coconut tree disease dataset.

## 3 Experimental Design, Materials, and Methods

### 3.1 Experimental design

The dataset for Coconut Tree Disease was obtained by taking pictures using the high-resolution rear cameras of a Samsung F23 5G Mobile. The data acquisition process consisted of two main steps, as summarized in Table 2.

**Step 1: *Image Acquisition (Duration: April to July):*** During this phase, we conducted field visits in daylight to capture images related to various coconut tree diseases. The main goal was to create a comprehensive collection of disease-related images.
**Step 2: *Image Pre-processing (Duration: July):*** In this step, we carefully reviewed the collected images and selected appropriate ones for the dataset. The selected images underwent pre-processing, which involved resizing, cropping, and enhancing them as needed using IrfanView Software. Pre-processing images in the dataset is important because it ensures uniformity in size, focuses on key features, and enhances image quality. This consistency helps machine learning models to better detect and classify diseases accurately.

**Table 2:** Data acquisition steps.

Sr. No.	Step	Duration	Activity
1.	Image Acquisition	April to July	During daytime field/farm visits to capture images.
2.	Image Pre-processing	July	The images appropriate for dataset were selected from gathered images and were pre-processed.

Overall, the data acquisition process involved capturing images during field visits and subsequently preparing them through pre-processing to include in the dataset.

### 3.2 Materials or specification of image acquisition system

The cameras used in the data acquisition process and the specifications of the captured images:

Samsung Galaxy F 23 5G Android Mobile:

• Make and Model: Samsung Galaxy F 23 5G (SM-E236B) Android Mobile.
• Rear Primary Camera: Equipped with a 50-megapixel (f/1.8) lens.
• Camera Sensor: Utilizes the Sony IMX 582 1/2" sensor.
• Battery: Comes with a 5000 mAh battery.

During the data collection process, efforts were made to adhere to standardized image acquisition practices, capturing each image using the rear cameras of a Samsung F23 5G Mobile known for its high-resolution imaging capabilities. This maintained consistency and quality throughout the dataset. Subsequently, the images were pre-processed using IrfanView Software, involving resizing, cropping, and contrast adjustments to enhance uniformity and highlight disease symptoms. The captured images were saved in JPG format and resized to a resolution of 768 × 1024 pixels. To ensure accurate labelling, a plant pathologists and agricultural specialists, from Rashtrapita Mahatma Gandhi Art's, Science College Nagbhir meticulously categorized each image with its respective disease category. This multi-step labelling process involved rigorous scrutiny and cross-validation by multiple experts to mitigate errors and enhance reliability. The validation of disease categories by domain experts not only added credibility to the dataset but also ensured the accurate representation of each disease's visual characteristics.

## 4 Method

The data for the coconut tree disease dataset was collected by visiting a farm in Kendur, Taluka- Shirur, District-Pune, India. Images were captured in various scenarios, including leaves in their natural environment and after being cut or separated from the plant. This allowed for a comprehensive representation of coconut tree diseases in different conditions.

Table 3 presents the distribution of images by various categories of coconut tree diseases in the dataset. The dataset consists of a total of 5798 images, with each category containing a different number of images. The categories include Bud Root Dropping, Bud Rot, Gray Leaf spot, Leaf Rot, and Stem Bleeding. These are relatively common diseases that can affect coconut trees. These diseases are known to occur in coconut cultivation regions and can lead to varying degrees of damage if not managed properly. The original format of the images is now accessible to the public through Mendeley [7]. The dataset empowers machine learning models to recognize and categorize diseases in coconut trees, streamlining disease monitoring and intervention efforts for improved plantation health. Coconut tree diseases can cause substantial financial losses by decreasing crop yield and necessitating costly disease management measures, affecting both revenue and expenses for coconut growers. Coconut tree diseases pose significant challenges to coconut cultivation regions worldwide, threatening livelihoods and economies. Machine learning-driven early detection using datasets like this can mitigate these challenges and promote sustainable coconut farming practices.

**Table 3:** Total number of images per category in the coconut tree disease dataset.

Disease Name	Total Images
Bud Root Dropping	514
Bud Rot	470
Gray Leaf spot	2135
Leaf Rot	1673
Stem Bleeding	1006
**Total**	**5798**

### 4.1 Evaluation framework

A robust evaluation is crucial to ascertain the dataset's efficacy in training accurate and reliable models for disease detection in coconut trees. We incorporate key metrics such as accuracy, precision, recall, and the F1-score to provide a holistic understanding of the models' capabilities. We utilized a dataset of coconut trees images categorized into five classes, representing different disease types. Prior to training, we employed the VGG16, ResNet50, and MobileNetV2 architectures, which are renowned for their capabilities in image recognition tasks [16]. We pre-processed the dataset, performed data augmentation to enhance model generalization, and fine-tuned the models for the specific classification task. Before training, the initial performance of the models was limited, with VGG16 achieving 0.2 % accuracy, ResNet50 achieving 0.4% accuracy, and MobileNetV2 achieving 0.25% accuracy. However, after training, substantial improvements were observed, with VGG16 achieving an accuracy of 88%, ResNet50 achieving 94% accuracy, and MobileNetV2 achieving 92% accuracy (Tables 4 and 5).

**Table 4:** Accuracy values of disease detection models.

Model	Accuracy before training	Accuracy after training on our dataset
**VGG16**	0.26%	88%
**ResNet50**	0.4%	94%
**MobileNetV2**	0.25%	92%

**Table 5:** Average performace values on machine learning models using 5 fold cross validation technique.

Model	Precision	Recall	F1 Score
**VGG16**	86.94%	87.52%	87.69%
**ResNet50**	92.25%	93.42%	93.88%
**MobileNetV2**	89.68%	90.45%	91.54%

The primary focus was directed towards the meticulous creation of a representative dataset, with the intention of mitigating class imbalance through specialized sampling techniques during subsequent model training. It is imperative to highlight that the reported results emanate from a comprehensive assessment encompassing 20 independent runs, a deliberate choice to bolster the robustness and reliability of our findings. Pertaining to the phenomenon of overfitting, it is noteworthy that the confusion matrix exhibited a discernible diagonal principal, indicative of a substantial proportion of correct predictions executed with high confidence. Furthermore, an in-depth analysis unveiled a disparity of less than 10% in recognition rates among distinct classes, providing further evidence to support the absence of overfitting.

Despite the notable improvements in accuracy post-training, the models exhibited signs of overfitting during testing. Overfitting occurs when a model performs well on the training data but struggles to generalize to unseen data, leading to diminished performance on the test set. This phenomenon was evident when evaluating the models on a separate test dataset. The models displayed higher accuracy on the training set compared to the test set, indicative of overfitting. The results indicate that while the models are capable of achieving high accuracy on the training data, their performance on unseen data remains suboptimal. Addressing overfitting is crucial to ensure that the models can make reliable predictions in real-world scenarios. Strategies such as incorporating regularization techniques, collecting additional diverse data, and exploring transfer learning approaches should be considered to mitigate overfitting and enhance model generalization. The dataset demonstrated the potential of deep CNNs for coconut leaf disease classification, achieving significant performance improvements through training. However, the challenge of overfitting during testing underscores the importance of further research to enhance the models' ability to generalize to new, unseen data. Addressing this challenge will contribute to the development of robust and reliable models for coconut disease detection, benefiting farmers and agriculture stakeholders.

### 4.2 Dataset applications

Through the application of advanced machine learning techniques, this dataset can markedly enhance disease diagnosis and management strategies. By training machine learning models on the dataset's diverse range of images depicting diseases like Bud Root Dropping, Bud Rot, Gray Leaf Spot, Leaf Rot, and Stem Bleeding, we can empower automated systems to rapidly and accurately identify disease symptoms. This capability enables timely intervention, as affected trees can be promptly treated or isolated, curbing the spread of diseases across plantations. Consequently, the dataset facilitates a paradigm shift towards proactive disease management, reducing the reliance on broad-spectrum pesticides and minimizing ecological impact [8], [9], [10], [11].

The dataset's utility extends beyond mere disease detection. Machine learning models, fuelled by the dataset's rich visual information, can predict disease outbreaks based on historical data and prevailing environmental conditions. This predictive capability allows farmers and plantation managers to make informed decisions regarding disease control measures, resource allocation, and crop rotation, ultimately optimizing agricultural practices and minimizing losses.

Machine learning-powered systems, utilizing the dataset's annotations, can guide targeted application of fertilizers, pesticides, and water resources. This optimization minimizes waste and environmental pollution, while ensuring that plants receive the necessary care tailored to their specific needs. This integration of data-driven insights and sustainable farming practices not only safeguards coconut plantations from disease-related challenges but also cultivates a resilient and ecologically conscious agricultural landscape [12,13].

The dataset holds promise for a range of practical applications beyond academic research, particularly within the agricultural sector and coconut plantation management. One such avenue involves its integration into automated monitoring systems, where machine learning algorithms harness the dataset to enable real-time disease detection and alerts for plantation owners and farmers. Collaborations with agricultural extension services could yield user-friendly platforms, allowing farmers to upload images of their coconut trees for swift disease diagnoses and tailored treatment recommendations. Additionally, machine learning models trained on the dataset could generate personalized disease management strategies, accounting for local conditions and best practices. The dataset's potential extends further to partnerships with technology companies, spurring the development of specialized hardware or software solutions for disease detection in coconut plantations. In essence, the ``Coconut Tree Disease Dataset'' stands poised to revolutionize disease monitoring, management, and knowledge dissemination, transcending academia to cultivate healthier and more sustainable coconut plantations. By harnessing the dataset's comprehensive images and disease annotations, machine learning can enhance precision agriculture approaches, optimizing irrigation, nutrient application, and crop protection. This synergy of advanced technology and agricultural expertise paves the way for a sustainable future, where data-driven decision-making mitigates disease impact, enhances productivity, and promotes environmentally conscious coconut plantation management [14,15].

## Limitations

The dataset is collected from a specific region, potentially limiting its applicability to other geographical areas with different disease prevalence or manifestations.

## Ethics Statement

Our study does not involve studies with animals or humans. Therefore, we confirm that our research strictly adheres to the guidelines for authors provided by Data in Brief in terms of ethical considerations.

## CRediT authorship contribution statement

**Sandip Thite:** Methodology, Writing – original draft. **Yogesh Suryawanshi:** Conceptualization, Writing – review & editing. **Kailas Patil:** Conceptualization, Supervision, Writing – review & editing. **Prawit Chumchu:** Writing – review & editing.

## Data Availability

Coconut Tree Disease Dataset (Original data) (Mendeley Data).
